# Anti- Japanese-Encephalitis-Viral Effects of Kaempferol and Daidzin and Their RNA-Binding Characteristics

**DOI:** 10.1371/journal.pone.0030259

**Published:** 2012-01-20

**Authors:** Ting Zhang, Zhiqiang Wu, Jiang Du, Yongfeng Hu, Liguo Liu, Fan Yang, Qi Jin

**Affiliations:** Institute of Pathogen Biology, Chinese Academy of Medical Science & Peking Union Medical College, Beijing, China; German Primate Center, Germany

## Abstract

**Background:**

New therapeutic tools and molecular targets are needed for treatment of Japanese encephalitis virus (JEV) infections. JEV requires an α-1 translational frameshift to synthesize the NS1' protein required for viral neuroinvasiveness. Several flavonoids have been shown to possess antiviral activity *in vitro* against a wide spectrum of viruses. To date, the antiviral activities of flavonol kaempferol (Kae) and isoflavonoid daidzin (Dai) against JEV have not been described.

**Methodology/Principal Findings:**

The 50% cytotoxic concentration (CC_50_) and 50% effective concentration (EC_50_) against JEV were investigated in BHK21 cells by MTS reduction. Activity against viral genomic RNA and proteins was measured by real-time RT-PCR and western blotting. The frameshift site RNA-binding characterization was also determined by electrospray ionization mass spectrometry, isothermal titration calorimetry and autodocking analysis. EC_50_ values of Kae and Dai were 12.6 and 25.9 µM against JEV in cells pretreated before infection, whereas in cells infected before treatment, EC_50_ was 21.5 and 40.4 µM, respectively. Kae exhibited more potent activity against JEV and RNA binding in cells following internalization through direct inhibition of viral replication and protein expression, indicating that its antiviral activity was principally due to direct virucidal effects. The JEV frameshift site RNA (fsRNA) was selected as a target for assaying Kae and Dai. ITC of fsRNA revealed an apparent K_b_ value for Kae that was nine fold stronger than that for Dai. This binding was confirmed and localized to the RNA using ESI-MS and autodock analysis. Kae could form non-covalent complexes with fsRNA more easily than Dai could.

**Conclusions/Significance:**

Kae demonstrates more potent antiviral activity against JEV than does Dai. The mode of action of Kae as an anti-JEV agent seems to be related to its ability to inactivate virus by binding with JEV fsRNA.

## Introduction

Viral infections are important public health problems worldwide, both in developed and developing countries, due to their morbidity and mortality. Japanese encephalitis virus (JEV) is a leading member of the mosquito-transmitted flavivirus family, and is mainly distributed in China, India and Southeast Asia, where it can cause the central nervous system disease with irreversible neurological damage in humans [1]. There are 30,000–50,000 cases of human Japanese encephalitis worldwide and 10,000–15,000 deaths each year. By some estimates, there may be as many as 75,000 cases each year [2]. JEV is also one of the main causes of infectious reproductive failure in swine, resulting in significant economic losses in the pig industry. This virus has a normal zoonotic transmission cycle between swine or birds and mosquitoes. Swine are the main amplifier hosts, from which infected mosquitoes transmit the virus to humans [3], [4].

The single, long open reading frame of the JEV genome encodes structural proteins (capsid, C), membrane (prM/M), and envelope (E) and non-structural proteins (NS1, NS2A, NS2B, NS3, NS4A, NS4B and NS5). JEV NS1 is involved in viral replication and regulation of the innate immune response. Recent research has identified that NS1' (a larger NS1-related protein), which plays a role in viral neuroinvasiveness, is the product of an α-1 ribosomal frameshift event that occurs at a conserved Y CCU UUU slippery heptanucleotide motif near the beginning of the NS2A gene, and is stimulated by a downstream RNA pseudoknot structure (a stem-loop structure, shown in Figure S1) [5], [6]. The stability of the stem-loop structure has been correlated with the efficiency of ribosomal frameshifting. Therefore, it is possible that small molecules that bind tightly to this sequence may interfere with the ability of the ribosome to engage the stable pseudoknot during frameshifting. Thus, the programmed translational frameshift site RNA (fsRNA) in the JEV serogroup might be an attractive target for designing anti-JEV drugs. Considering the current limited number of therapeutic options for JEV infection and no clearly effective antiviral agents, *in vitro* screening of potentially active compounds is a useful step in the preclinical development of novel drugs.

Plant-derived flavonoids and dietary isoflavones, a large group of naturally occurring phenylchromones found in fruits, vegetables, tea, soy foods, and herbs, have been shown to possess potential therapeutic benefits in a variety of viral infections using both *in vitro* or *in vivo* models [7], [8]. The predominant isoflavones found in soybeans are the β-glycoside forms (genistin, daidzin and glyctin) of genistein, daidzein and glycitein, which are not bioavailable [9], [10]. Upon ingestion, small intestinal brush border membrane enzymes and bacterial β-glycosidases remove the glycoside group, after which the isoflavones are readily absorbed and become bioactive. By far, genistein is the most studied soy isoflavone, and it has been shown to inhibit the infectivity of enveloped or non-enveloped viruses, as well as single-stranded (ss) or double-stranded (ds) RNA or DNA viruses [8]. To the best of our knowledge, no study has investigated the antiviral properties of daidzin *in vivo* or *in vitro*.

Kaempferol, a polyhydric flavonol, is isolated from various plant sources including tea, broccoli, delphinium, witch-hazel, grapefruit, Brussel sprouts, apples, and medicinal herbs. Kaempferol has been reported to exhibit activity against herpes simplex virus, influenza viruses (H1N1 and H9N2) and hepatitis B virus under *in vitro* conditions [11], [12], but until now, the antiviral properties of kaempferol against JEV have not been described. For these reasons, a detailed study of dietary kaempferol and daidzin activity against JEV in BHK21 cells could help to understand better their antiviral mechanisms and their application in dietary prevention and treatment of virus infection.

Comprehensive studies on the nature of interactions between small molecules and DNA/RNA are of fundamental importance for biomedical and pharmaceutical sciences. For example, several classes of mRNA domains, collectively referred to as riboswitches, have been shown to serve as RNA genetic control elements that sense the concentrations of specific metabolites (i.e. acting as direct sensors of chemical compounds) [13], [14]. Therefore, the conserved sequence of JEV frameshift site stem-loop was selected to characterize the interactions between RNA and kaempferol and daidzin, using electrospray ionization mass spectrometry (ESI-MS) and isothermal titration calorimetry (ITC). The two ligand molecules were docked into the RNA using AutoDock 4.0 program.

## Results

### Cellular Toxicity of Kae and Dai in BHK21 Cells

**Figure 1:**
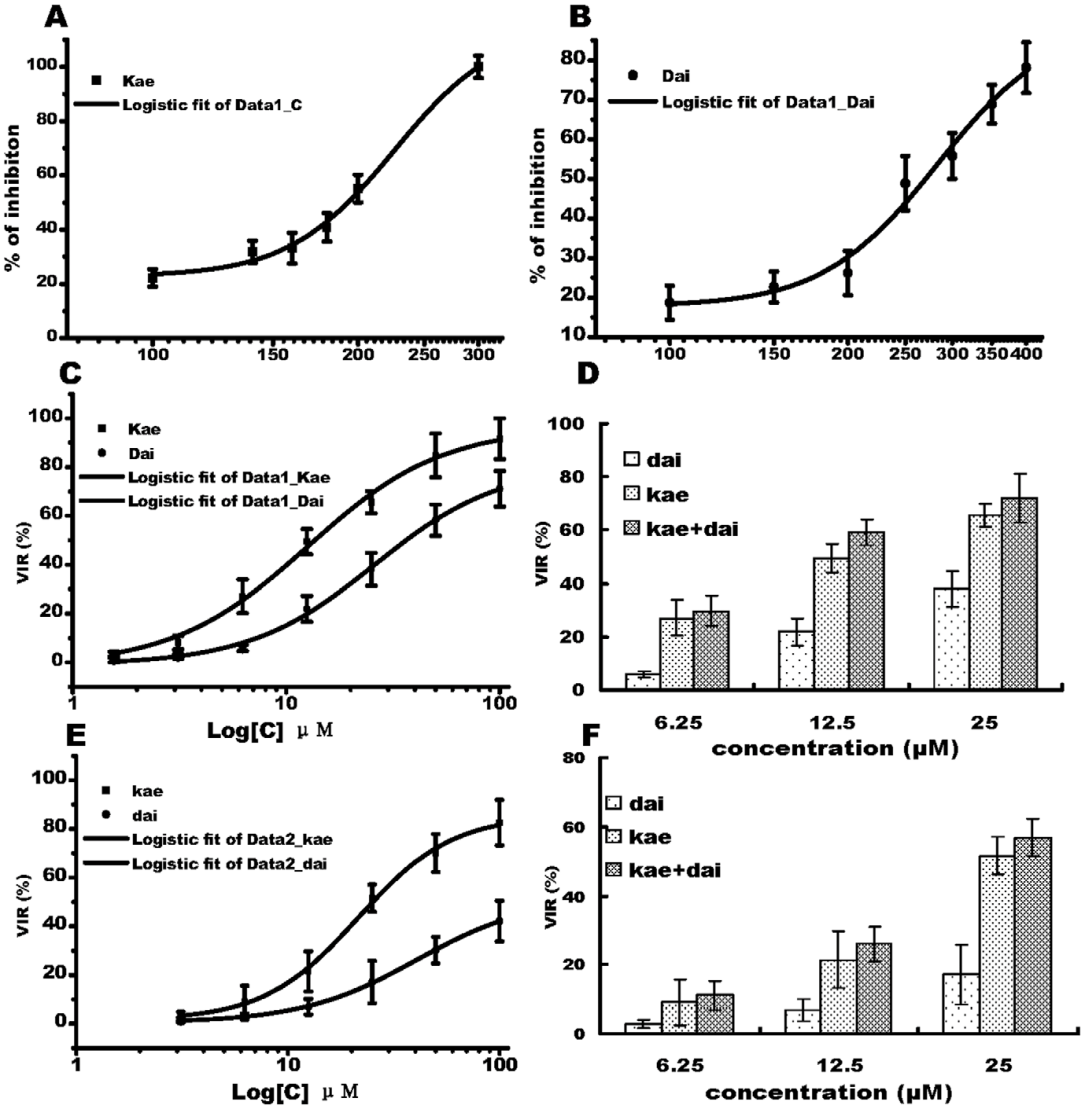
Toxicity of Kae and Dai on BHK21 cells and their effects on JEV infection. (A) Growth inhibition curve of Kae on BHK21 cells for 72 h. CC_50_ was 230 µM. (B) Growth inhibition curve of Dai on BHK21 cells for 72 h. CC_50_ was 270 µM. (C) Anti-JEV effects of Kae and Dai when cells were pretreated for 2 h before infection with 0.1 MOI JEV for 72 h. EC_50_ for Kae and Dai at 72 h were 12.6 and 25.9 µM, respectively. (D) Comparison of anti-JEV effects of combination treatment with Kae and Dai when cells received combined pretreatment for 2 h, and were then infected with 0.1 MOI JEV for 72 h. (E) Anti-JEV effects of Kae and Dai when cells were infected with 0.1 MOI JEV for 2 h and then treated with Kae or Dai for 72 h. EC_50_ was 21.5 µM for Kae and 40.4 µM for Dai. (F) Comparison of anti-JEV effects of combination treatment with Kae and Dai when cells were infected with 0.1 MOI JEV for 2 h followed by combination treatment for 72 h. The data represent the means for five replicate samples of three separate experiments. Kae (black square) or Dai (black circle).

Cellular toxicity of Kae and Dai were assessed in BHK21 cells using the cell MTS reduction assay. When confluent monolayers were exposed to Kae or Dai at concentrations of 50–400 µM for 72 h at 37°C, both agents exhibited a concentration-dependent inhibition of cell growth compared to controls (Figure 1A, B). The CC_50_ of Kae and Dai against BHK21 cells was 230 and 270 µM, respectively. The maximum non-toxic doses were determined at 180 and 225 µM respectively, and these were used as the highest doses in the antiviral assays. For comparison, 7-hydroxyflavone did not show obvious growth inhibitory effects (data not shown).

#### Antiviral Activity of Kae and Dai Against JEV in BHK21 Cells

The antiviral activity of Kae or Dai against JEV was also evaluated by MTS assay. When cells were pretreated with Kae or Dai or their combination for 2 h, and then infected with 0.1 MOI JEV for 72 h at 37°C, both Kae and Dai exhibited dose-dependent inhibition of JEV (Figure 1C, D). The EC_50_ for Kae was 12.6 µM against JEV and 70% inhibition at 25.7 µM; whereas for Dai, EC_50_ and 70% inhibition values were 25.9 and 92.4 µM, respectively (Figure 1C). Viral inhibition rate (VIR) of combined Kae and Dai at a concentration of 12.5 µM was 59.17%, whereas VIR was 49.5% for Kae and 21.9% for Dai at the same concentration (Figure 1D). When cells were infected with 0.1 MOI JEV for 2 h, and then treated with Kae, Dai or their combination (Figure 1E, F) for 72 h, the EC_50_ and 70% inhibition for Kae were 21.5 and 45.8 µM, respectively, whereas the EC_50_ for Dai was 40.4 µM (Figure 1E). The VIR of Kae, Dai and their combination was 21.5, 6.9 and 25.9%, respectively, at a concentration of 12.5 µM (Figure 1F). The results demonstrated that Kae was a more efficient inhibitor of JEV than Dai was. Moreover, the combination of Kae and Dai was more effective against JEV infection than either agent alone. In contrast, 7-hydroxyflavone, which has only one hydroxyl moiety, had no anti-JEV activity.

**Figure 2:**
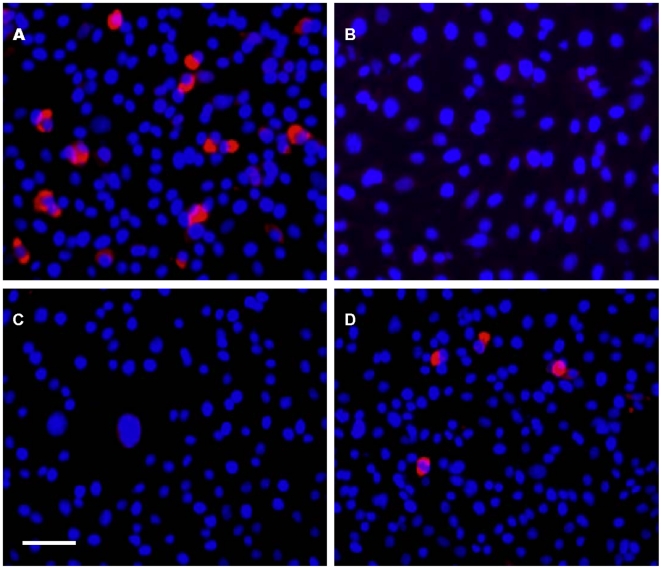
Antiviral effects of Kae and Dai on JEV in IFA. (A) Virus-infected BHK21 cells without Kae or Dai. There were many infected cells (red). (B) Healthy control BHK21 cells showed no positive fluorescence reaction. (C) Virus-infected BHK21 cells with treated with Kae. There was a marked reduction in infected cells. (D) Virus-infected BHK21 cells treated with Dai. Bar: 100 µm.

When the anti-JEV activity of Kae and Dai was tested by immunofluorescence assay (IFA), red fluorescence was observed in early virus-infected BHK21 cells at 24 h (Figure 2A), but not in healthy control cells (Figure 2B). The number of fluorescence-positive cells was reduced significantly in cells treated with 25 µM Kae or Dai compared to the mock-infected group (Figure 2C, D).

### Inhibitory Effects of Kae and Dai on JEV mRNA and E and NS1/NS1' Protein Expression

**Figure 3:**
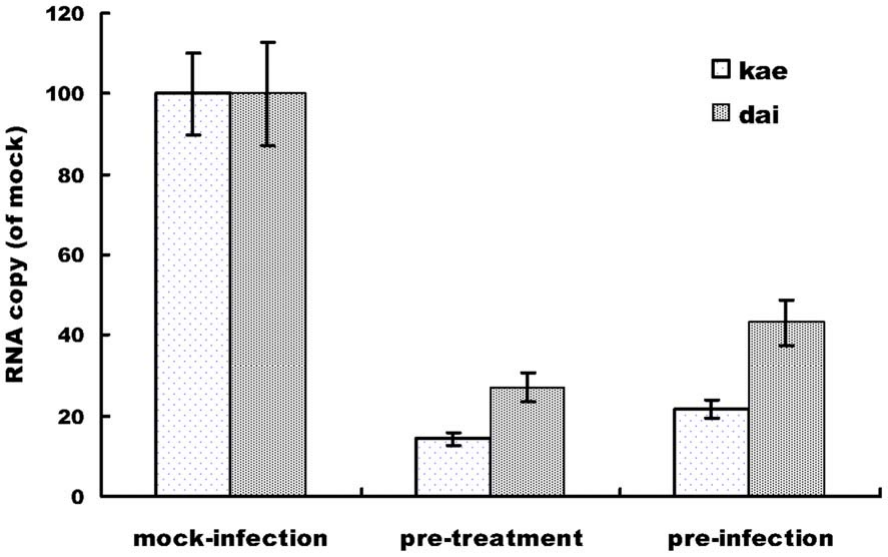
Comparison of the inhibitory effects of 25 µM Kae and Dai on JEV mRNA expression at 48 h. (A) mRNA expression level of JEV-infected cells (mock infection) was defined as 100%. The amount of viral RNA was normalized by the amount of cellular β-actin transcripts. Mock infection: infection alone without compound treatment; pretreatment: cell monolayers were pretreated with Kae or Dai for 2 h, and then infected for 48 h; pre-infection: cell monolayers were infected with 0.1 MOI of JEV for 2 h and then treated with Kae or Dai for 48 h. The data represent the means for five replicate samples of three separate experiments.

Total RNA was extracted from each group of cells treated for 48 h. JEV mRNA levels were determined using SYBR Green real-time RT-PCR (relative quantification). Kae and Dai inhibited JEV mRNA transcription of target genes by >14.2% and 27.1%, respectively, at 48 h pre-treatment group, compared with the mock-infected group. In pre-infection group, viral RNA replication was inhibited by 21.6% by Kae and 43.1% by Dai, compared with the mock-infected group (Figure 3). These findings show that Kae and Dai play critical roles in inhibition of JEV RNA replication.

**Figure 4:**
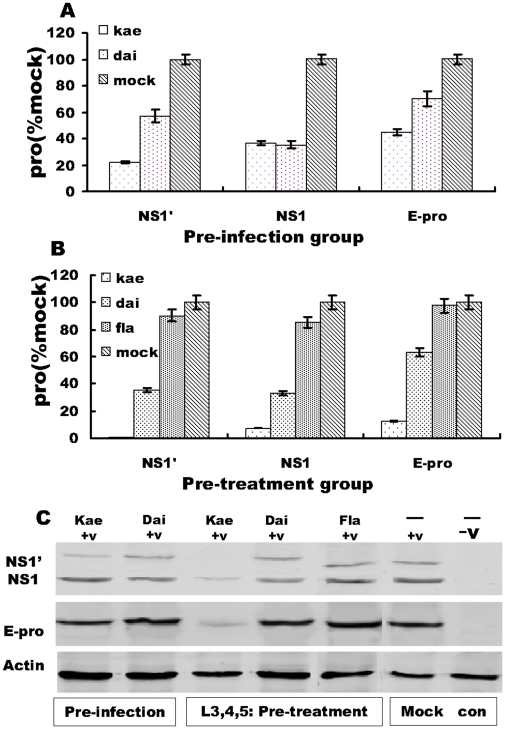
Comparison of the inhibitory effects of 25 µM Kae and Dai on JEV E, NS1 and NS1' protein expression at 48 h. (A) Histogram of JEV E, NS1 and NS1' protein expression levels in cells infected and then treated with Kae or Dai. (B) Histogram of viral E, NS nd NS1' protein expression levels in cells pretreated with Kae or Dai before infection. (C) Representative western blots of E, NS1 and NS1' protein expression. The E, NS1 and NS1' protein level in JEV-infected cells (mock infection) was defined as 100%. The amount of E, NS1 and NS1' proteins was normalized to the amount of cellular β-actin. Mock infection: infection alone without compound treatment; pretreatment: cell monolayers pretreated with Kae or Dai for 2 h, and then infected for 48 h; preinfection: cell monolayers infected with 0.1 MOI of JEV for 2 h and then treated with Kae or Dai for 48 h, and 7-hydroxyflavone as treatment control. Data shown were from three independent experiments with the mean and standard errors.

The effect of 25 µM Kae/Dai in each groups on viral E and NS1/NS1' proteins was detected by immunoassay with the Li-COR Odyssey system and quantified using Odyssey infrared imaging software (Figure 4). When compared with the mock-infection group, E, NS1 and NS1' protein expression was inhibited by >44.8%, 36.8% and 22.1% by Kae, and >70.1%, 35.3% and 57.2% by Dai, respectively, at pre-infection group (Figure 4 A, C). When cells were pretreated before JEV infection (pre-treatment group), E, NS1 and NS1' protein expression was inhibited by >12.9%, 7.1% and 1.2% by Kae, and >63.2%, 33.3% and 35.5% by Dai, respectively, when compared with the mock-infection group (Figure 4B, C). It was clear that Kae pre-treatment before JEV infection inhibited viral protein expression more than treatment after infection, and more than Dai administered before or after infection. Moreover, under the same treatment conditions, the expression level of NS1 and NS1' proteins was lower than that of E protein in mock-infected cells. It may be a differential feature of the virus stains used in the experiment. However, expression of NS1 and NS1' was clearly suppressed in cells treated with Kae or Dai.

No changes in E, NS1 and NS1' protein expression was observed in cells treated with 7-hydroxyflavone pre-treatment group (Figure 4B, C).

#### Kae and Dai Membrane Translocation and JEV RNA Binding in Cells

**Figure 5:**
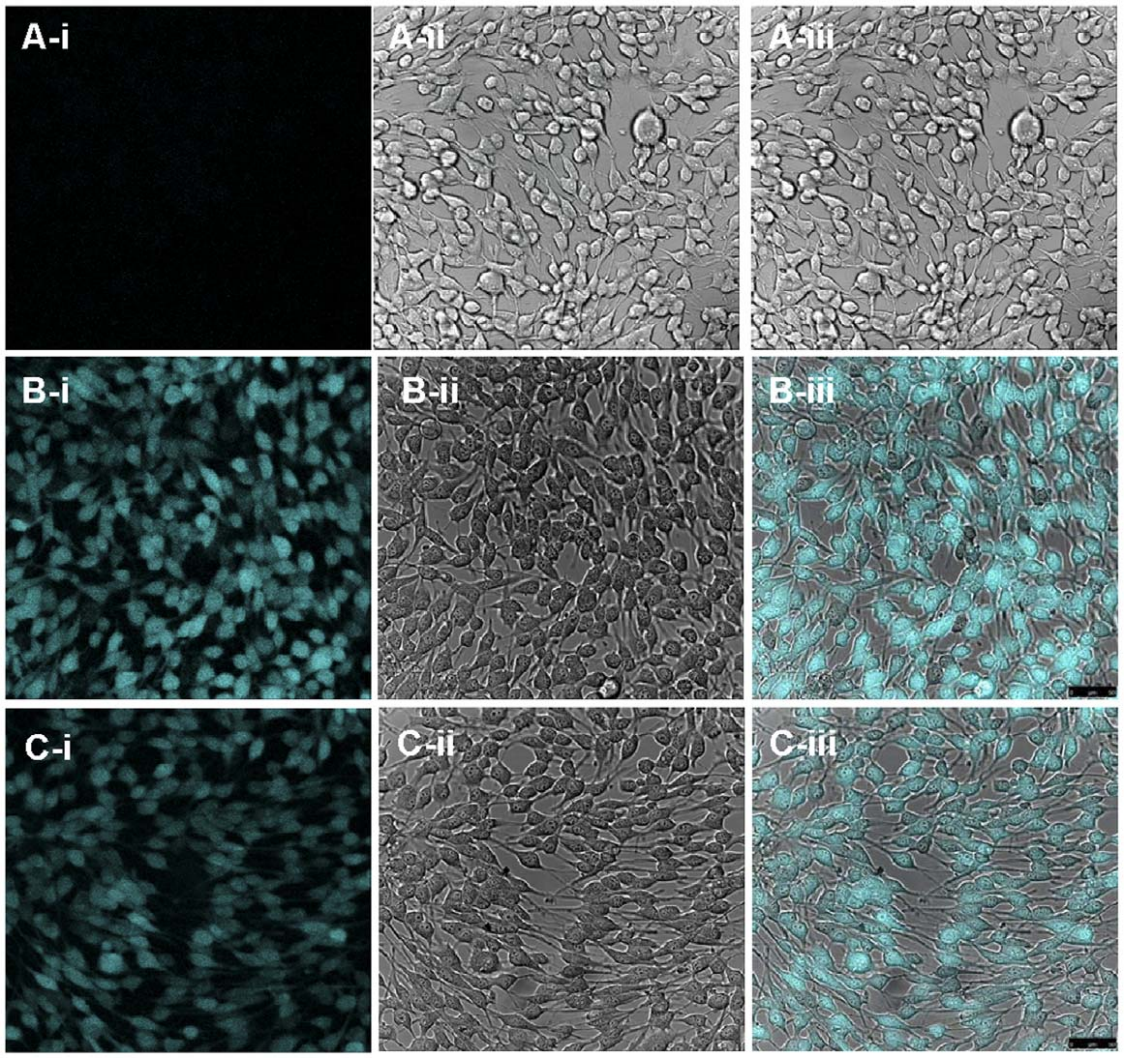
Internalization of Kae in BHK21 cells. Twenty-four-hour-infected cell monolayers and healthy cells grown on confocal dishes were incubated with 50 µM kae for 2 h. Fluorescence was detected at 480–500 nm after excitation at 405 nm with an argon laser. Images of a single middle layer from z-stacks are shown. (A) Cells infected with JEV for 24 h and then treated with 0.1% DMSO for 2 h; (B) cells infected with JEV for 24 h and then treated with Kae for 2 h; (C) cells treated only with Kae for 2 h. Images represent results from at least three individual experiments. Bar: 50 µm.

To determine whether Kae and Dai were able to reach the cytosol, and their binding characteristics with JEV RNA, the intrinsic fluorescent properties of flavonoids [15] were utilized to visualize their presence within the cells. Following 2 h incubation with Kae (50 µM), Dai (100 µM) or control solvent in uninfected cells or cells that had been infected for 24 h, a series of images in the z dimension at 0.4 µm intervals were obtained through the cell membrane and cytosol. A single image of a layer from the middle of the series illustrated internalization of Kae (Figure 5). No auto-fluorescence was observed in the mock-infection group (Figure 5A). In Kae-treated cells, fluorescence was intense and spread across the entire cell (Figure 5B, C). The intrinsic fluorescence of Kae observed in cells that had been infected for 24 h before treatment (Figure 5B) was more intense than that in uninfected cells (Figure 5C), which suggests that Kae inhibits viral activity by binding to viral RNA within the cell cytosol following internalization. However, no fluorescence was detected in either Dai- or 7-hydroxyflavone-treated infected or uninfected cells at any time point (data not shown), indicating that Dai and 7-hydroxyflavone may have lower transmembrane permeability and/or intrinsic fluorescence.

### Stoichiometry of Kae/Dai and fsRNA of ESI-MS

**Figure 6:**
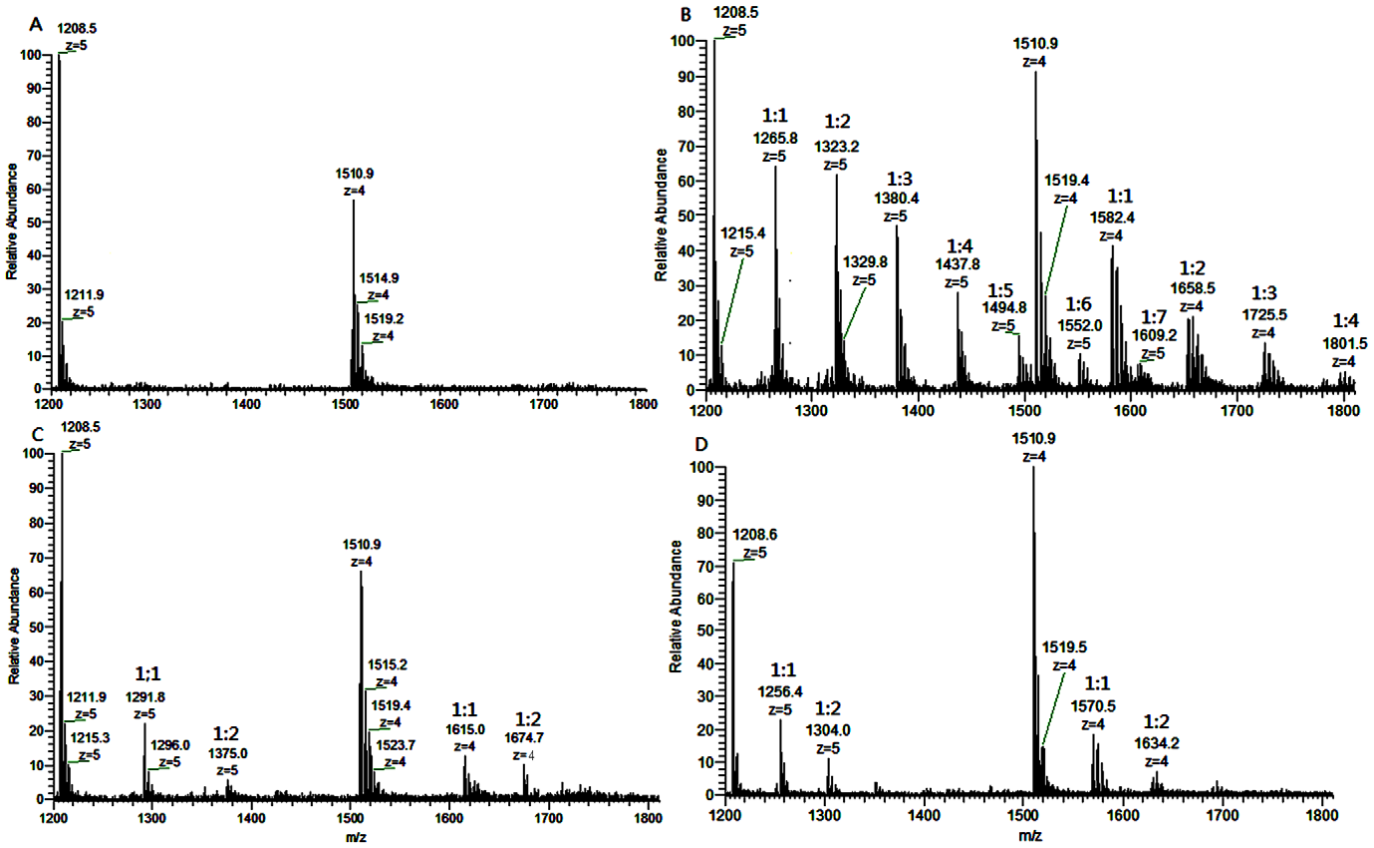
ESI-MS full-scan spectra of fsRNA1 (R1, MW, 6048 Da) and its mixtures with 50 µM Kae and Dai. (A) Mass spectrum of 5 µM RNA alone. RNA solution was prepared by mixing equal volumes of methanol and a 10 µM (150 mM ammonium acetate) solution of RNA; (B) Mass spectrum offsRNA1 with Kae. The solution was prepared by mixing equal volumes of 50 µM methanol solution of Kae and 10 µM (150 mM ammonium acetate) solution of fsRNA1; (C) Mass spectrum offsRNA1 with Dai. The solution was prepared by mixing equal volumes of 50 µM methanol solution of Dai and 10 µM (150 mM ammonium acetate) solution of fsRNA1; (D) Mass spectrum offsRNA1 with 7-hydroxyflavone. The solution was prepared by mixing equal volumes of 80 µM methanol solution of 7-hydroxyflavone and 10 µM (150 mM ammonium acetate) solution of fsRNA1. fsRNA1 alone, and 1∶1, 2∶1, 3∶1, 4∶1, 5∶1, 6∶1 and 7∶1 complexes are indicated.

**Figure 7:**
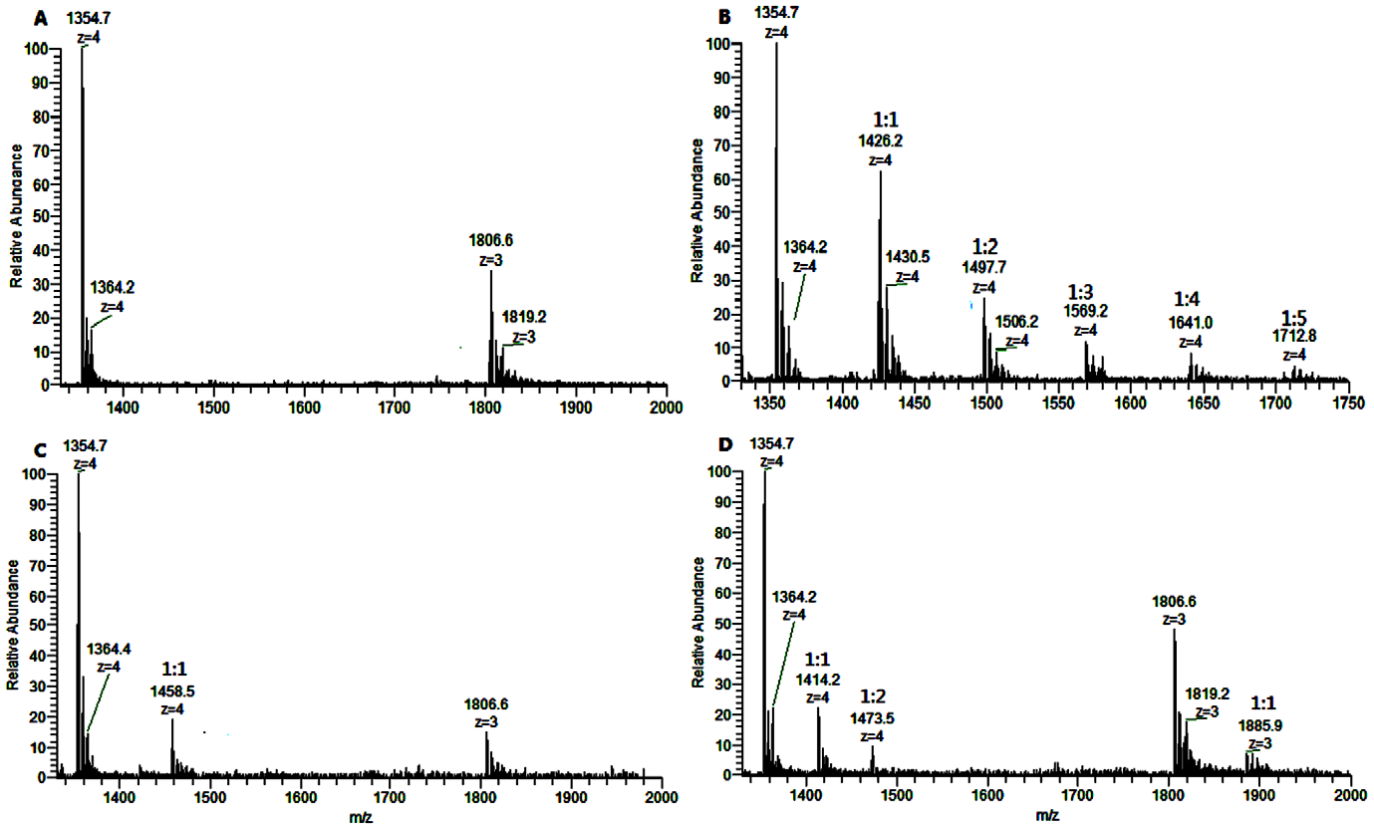
ESI-MS full-scan spectra of fsRNA2 (R2, MW, 5423 Da) (5 µM) and its mixtures with 50 µM Kae and Dai. (A) RNA alone; (B) with Kae; (C) with Dai; (D) with 7-hydroxyflavone. The solution was prepared as described above. fsRNA2 alone, and 1∶1, 2∶1, 3∶1, 4∶1 and 5∶1 complexes are indicated.

ESI-MS spectra are used to determine the stoichiometry and affinity of flavones for DNA/RNA in the presence of 150 mM monovalent ammonia ions [16]. Figure 6A shows an ESI mass spectrum obtained from 5 µM fsRNA1 solution. The spectrum showed a distribution of multiple peaks, with a number of charges, z = 5 and z = 4. There were two groups of signals corresponding to the free fsRNA1 (1208.5^5−^, 1211.9^5−^ and 1216.1^5−^) for the charge state z = 5 and (1510.9^4−^, 1514.9^4−^ and 1519.2^4−^) for the charge state z = 4. The average molecular mass measured from the main peaks was 6048, 6060 and 6072 Da respectively. Figure 6B and C also shows an ESI mass spectrum obtained from the mixed solution of 50 µM Kae/Dai and 10 µM fsRNA1. Besides the expected multiple negative ions, a mixture of 1∶1 (1265.8^5−^), 2∶1 (1323.2^5−^), 3∶1 (1380.4^5−^), 4∶1 (1437.8^5−^), 5∶1 (1494.8^5−^), 6∶1 (1552.0^5−^) and 7∶1 (1609.2^5−^) ligand–fsRNA1 complexes for the charge state z = 5 and 1∶1 (1582.4^4−^), 2∶1 (1658.5^4−^), 3∶1(1725.5^4−^), 4∶1 (1701.5^4−^) for the charge state z = 4 complexes were detected in the case of Kae (Figure 6B), whereas only 1∶1 (1291.8^5−^ and 1615.0^4−^) and 2∶1 (1375.0^5−^ and 1674.7^4−^) ligand–fsRNA1 complexes were observed with Dai (Figure 6C). Figure 7 shows the ESI-MS spectra containing 10 µM fsRNA2 with 50 µM Kae/Dai. The ions corresponding to fsRNA2 and the complexes appeared in the spectra with a charge z = 4; the largest stoichiometric ratio was 5∶1 (1712.8^4−^) for Kae–fsRNA2 complexes and 1∶1 (1658.5^4−^) for Dai, which suggests that Kae and Dai can both form adducts with fsRNA1 or fsRNA2 in a diverse stoichiometric ratio. These ESI-MS results demonstrated different binding abilities of Kae and Dai. To make semi-quantitative comparisons between Kae and Dai, the affinity of the ligands for fsRNA1 and fsRNA2 was characterized by the concentration of bound ligand for each RNA molecule, as described by Rosu et al. [16]. The concentration of Kae bound to fsRNA1 and fsRNA2 was 1.901 µM and 1.213 µM for each RNA molecule, which was higher than that of daidzin (0.686 µM per fsRNA1 and 0.452 µM per fsRNA2). Thus, Kae has higher binding affinity to fsRNA1 and fsRNA2 than Dai in the presence of ammonia ions.

As a control, we selected a 7-hydroxyflavone displaying no anti-JEV activity to test its affinity to fsRNA1 and fsRNA2. Increasing the ratio of 7-hydroxyflavone∶fsRNA1 to 80∶1 allowed the detection of complexes of 2∶1 7-hydroxyflavone∶fsRNA1/fsRNA2 complexes (Figures 6D, 7D), but it had a lower relative abundance when compared with Kae∶fsRNA1/fsRNA2 complexes (Figures 6B, 7B). We verified that Kae had higher binding affinity to the fsRNA1 and fsRNA2 than Dai and 7-hydroxyflavone had in the presence of ammonia ions.

### Characterization of Ligand–RNA Interaction by ITC_200_ Analysis

**Figure 8:**
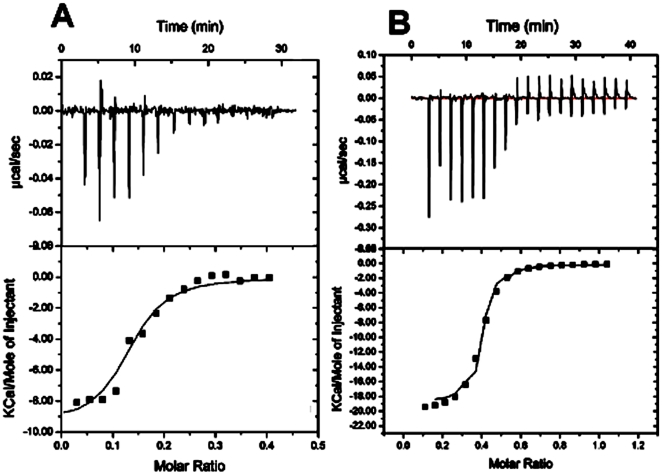
ITC profile for the binding of fsRNA3 (R3) to Dai (A) and Kae (B) at 25°C in 50 mM Tris–HCl buffer containing 150 mM NaCl and 0.1% DMSO (pH 7.3). The top panels represent the raw data for sequential injection of RNA into the ligands (curves on the bottom) and RNA dilution control (curves offset for clarity). The bottom panels show the integrated heat data after correcting for the heat of dilution of RNA against the molar ratio of Kae to RNA. The data (open squares) were fitted to a one-site model and the solid lines represent the best fit of the data.

The interactions of Kae and Dai ligands with fsRNA3 were studied by the ITC_200_ method. The fsRNA3 solution was placed at room temperature for 24 h to obtain a stable stem-loop structure before the tests. The thermodynamic parameters for the binding of fsRNA3 obtained at 25°C are summarized in Figure 8. The ITC data for Dai binding to fsRNA3 (Figure 8A) yielded a K_b_ of 9.02±1.06×10^6^ M^−1^, a ΔH = −9.5±1.19 kcal mol^−1^, a ΔS of −0.05 cal mol^−1^ K^−1^, and a binding site size of eight nucleotides. The value for Kae with fsRNA3 (Figure 8B) had an apparent K_b_ of 84.2±4.24×10^6^ M^−1^, which was ninefold stronger than that of Dai; an enthalpy value of ΔH = −15.95±0.47 kcal mol^−1^, which decreased obviously compared with that of Dai, and also displayed a large unfavorable entropic component (−17.2 cal mol^−1^ K^−1^) and a binding site size of 2.7 nucleotides. This entropy was ∼34-fold greater in magnitude than that observed for Dai, suggesting that Kae gave rise to changes in fsRNA3 conformation compared with Dai. In contrast, there were no constant thermodynamic parameters between 7-hydroxyfalvone and RNA (data not shown). Perhaps the use of simpler models is not suitable for 7-hydroxyflavone binding because the thermogram fit values were unacceptable.

#### Characterization of Kae/Dai Binding to RNA by Docking Study

**Figure 9:**
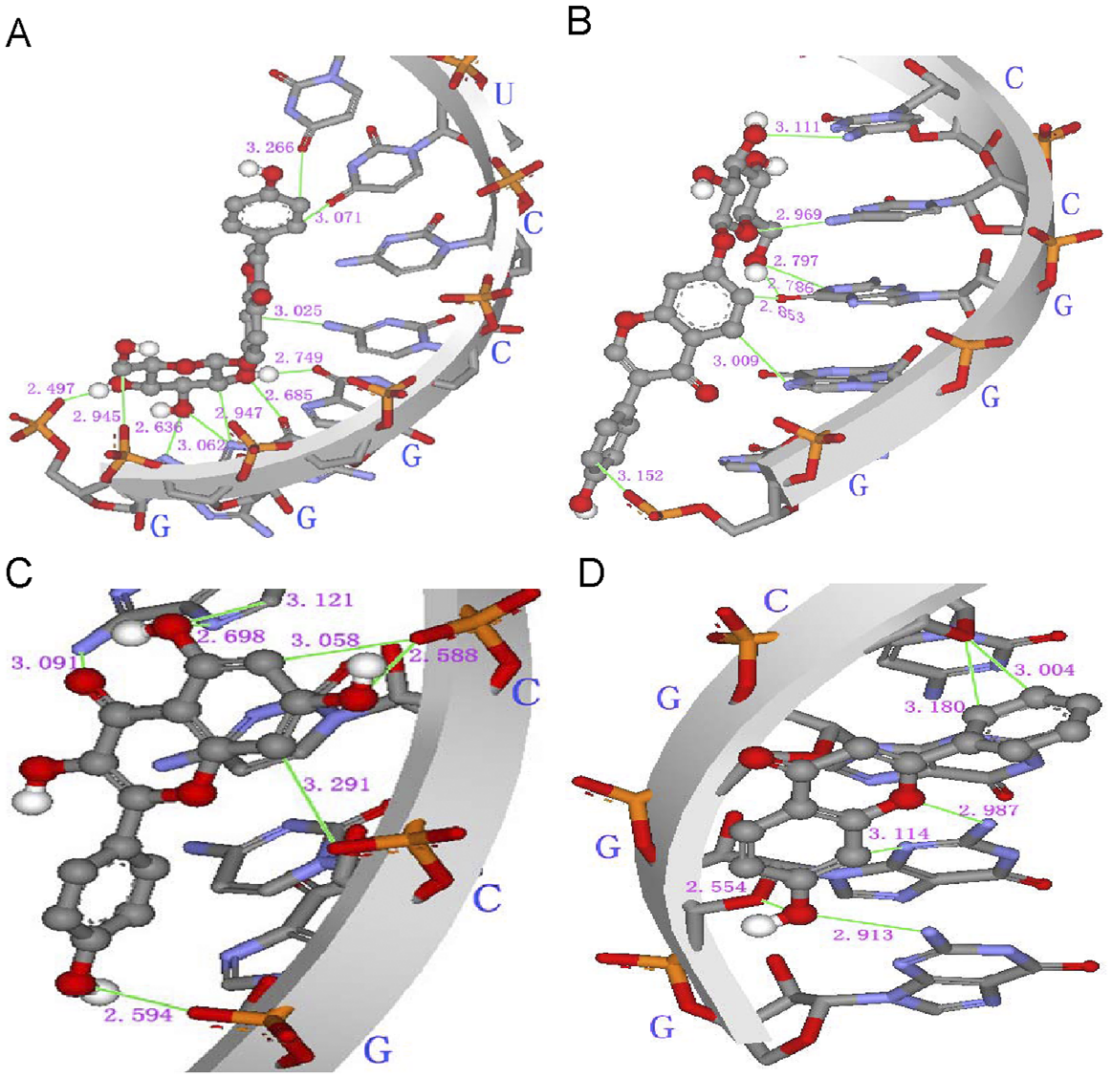
Top-ranked docking conformation for Dai (a) and Kae (b) with fsRNA and hydrogen bonds between the ligands and fsRNA. (A) R1a; (B) R2a; (C) R1b and (D) R2b.

The computed binding energy (BE) and corresponding energy difference value (ΔE) for each group are listed in Tables 1 and 2. A negative value of BE means that the corresponding RNA-ligand complex is energetically stable; the more negative the BE is, the more stable the complex is. These quantities reflected the ability of RNA to bind to Dai (a) and Kae (b). From three fsRNA docking results (Table 1), the BE of Kae complexes with each RNA molecule was always lower than that of Dai complexes with each corresponding RNA molecule. Moreover, the complex of Kae with R3 molecule had the lowest binding energy in all RNA docking results. When Kae was docking into R4, with no base ‘G’ in sequence, BE of the Kae–R4 complex increased to −4.14 kcal mol^−1^, and elevated by 0.9 kcal mol^−1^ compared to that of the Kae–R1 complex (−5.04 kcal mol^−1^). This indicated that base G might influence the binding ability between Kae and RNA. The top ranked dock conformation for Kae and Dai with fsRNA is presented in Figure 9. Visualization of the dockings reveals the top ranked conformation and binding sites which contain a sequence of three base (CGG or CCG) when ligands are docked into the fsRNA.

**Table 1:** The binding energies and corresponding ΔE^a^ upon the inclusion complex of fsRNA-ligands (a and b).

Ra complex	BE(kcal.mol^−1^)	Rb complex	BE(kcal.mol^−1^)	ΔE^d^(kcal.mol^−1^)
R1a	−3.18	R1b	−3.64	0.46
R2a	−3.46	R2b	−3.70	0.24
R3a	−3.89	R3b	−5.04	1.15

BE: binding energy upon complex; ΔE^d^: Energy difference value between Rb and Ra complex; Dai(a); Kae(b); fsRNA1(R1); fsRNA2(R2); fsRNA3(R3); Ra complex: ligand a binding with R1,R2 and R3, respectively; Rb complex: ligand b binding with R1,R2 and R3, repectively.

**Table 2:** The binding energies and corresponding ΔE^a^ upon the inclusion complex of dsRNA-ligands (a and b).

acomplex	BE(kcal.mol^−1^)	bcomplex	BE(kcal.mol^−1^)	ΔE^d^(kcal.mol^−1^)
U1a	−3.94	U1b	−4.83	0.89
U2a	−3.91	U2b	−5.22	1.31
U3a	−3.63	U3b	−5.17	1.54
U4a	−3.88	U4b	−5.02	1.14
N1a	−4.46	N1b	−5.64	1.18
N2a	−4.26	N2b	−5.32	1.06
N3a	−4.05	N3b	−5.35	1.30
N4a	−3.96	N4b	−5.29	1.33

BE: binding energy upon complex; ΔE^d^: Energy difference value between ^a^complex and ^b^complex; Dai(a); Kae(b); U1–U4: dsRNA with UUU triplets; N1–N4: dsRNA without UUU triplets;

a complex: ligand a binding with U1–U4 and N1–N4, respectively;

b complex: ligand b binding with U1–U4 and N1–N4, respectively.

**Figure 10:**
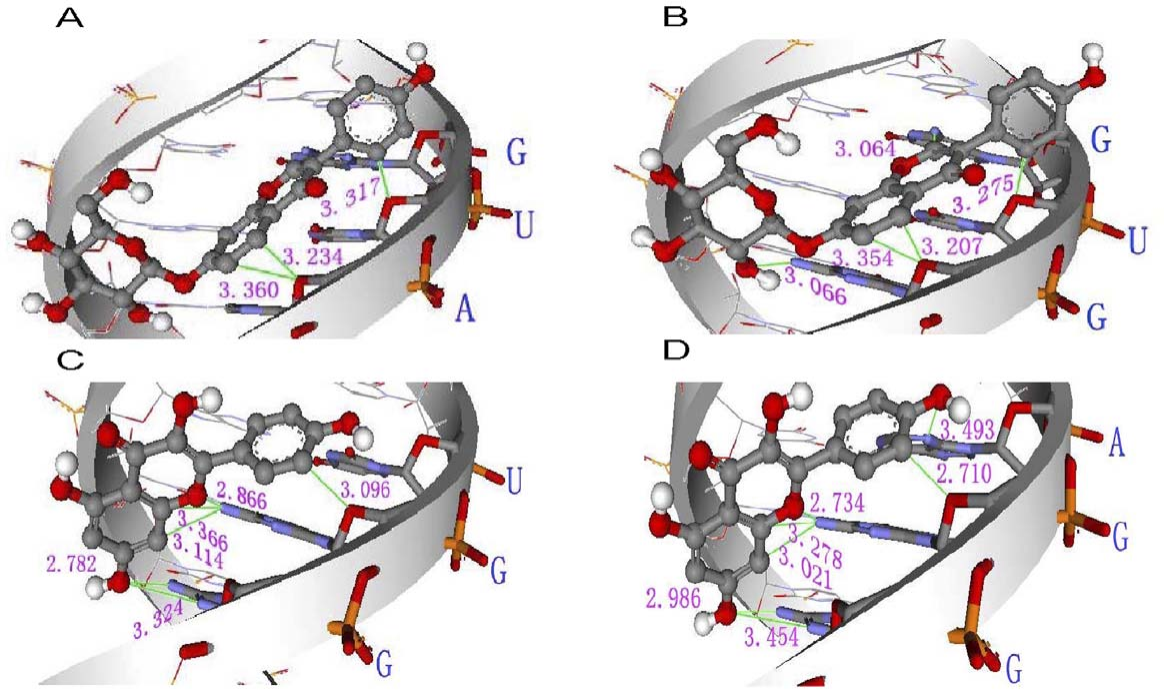
Top-ranked docking conformation for Dai (a) and Kae (b) with RNA duplex and hydrogen bonds between the ligands and dsRNA. (A) U1a; (B) N1a; (C) U2b; and (D) N1b.

In double stain RNA (dsRNA) docking results, the inclusions U1a, N1a and N1b had the highest probabilities and largest BE. The lowest binding energy and frequencies characterizing inclusion of Dai and Kae into RNA molecules are depicted separately in Table 2. Most of the complexes of Kae with RNA molecules with a UUU triplet had a BE that was 1 kcal mol^−1^ lower than that of RNA duplexes that contained UUU triplets with Dai. We obtained similar results for the inclusion complexes of RNA without UUU with Kae and Dai. Visualization of the dockings reveals that the ligands are docked into the minor groove of RNA duplexes. The top ranked dock conformation for Kae and Dai with RNA duplexes and the detailed intermolecular hydrogen bonds are presented in Figure 10. Dai or Kae remained almost parallel to the double strand of the minor groove of the RNA molecule, and there were many hydrogen bond interactions between RNA and the ligands, as described in the systematic docking studies. These observations clearly showed that the number of hydrogen bonds for the N1b system was larger than for the other dsRNAs. There were potentially five and seven hydrogen bonds formed between the two ligands and RNA molecules for N1a and N1b systems, respectively. The main contributions of hydrogen bonds in N1a involved binding between C–H groups or O–H groups of ligand A; and O and N atoms of the G7, U8 and G9 bases of the corresponding N1 sequences. However, N1b involved more hydrogen bonding events by C–H or O–H groups of ligand B and N–H groups or N atoms of the A13, G14 and G15 bases of N1 sequences. The O–H groups of the linking regions in B play an important role in the interaction by functioning as hydrogen bond donors to the N atom of the nucleobase located on the floor of the minor groove of RNA duplexes.

## Discussion

Flaviral infections, including JEV, are an important public health problem in mainland China due to the limited therapeutic options and unsatisfactory outcomes. Therefore it is necessary to screen anti-JEV molecules for the development of novel antiviral therapies. Usually, cell culture monolayers infected with different DNA or RNA viruses are the most frequently used *in vitro* models for determining antiviral activity of natural compounds [17]. Similarly, in the present study, the putative effect of Kae and Dai against JEV was studied in BHK21 cells. Anti-JEV activity of Kae and Dai in BHK21 cells was analyzed by MTS reduction assay differed markedly from each other in different infection time points, although both agents showed dose-dependent anti-JEV activity. When cells were pretreated for 2 h and then infected with JEV infection and incubated for 72 h at 37°C, EC_50_ against JEV for Kae and Dai was 12.6 µM (SI = 18.2, SI = CC_50_/EC_50_) and 25.9 µM (SI = 10.4), respectively. The antiviral activity of Kae and Dai may have been due to inhibition of viral infection or a reduction in viral replication by modulating cellular functions through a number of mechanisms. We next studied viral inactivation in cells that were infected with JEV for 2 h before treatment with Kae or Dai. EC_50_ for Kae and Dai was 21.5 and 40.4 µM, respectively, suggesting that they inhibited events downstream of viral entry. MTS and IFA results both revealed that Kae had a more effective and higher SI value (15.8) than Dai did, suggesting that Kae is a more potent antiviral agent against JEV, with no toxicity to BHK21 cells. Furthermore, combination of Kae and Dai was more efficient against JEV infection, suggesting a possible synergy in the antiviral properties of flavonoid and isoflavonoid mixtures.

To further characterize the effects of Kae/Dai treatment on the cellular response to JEV infection, we investigated their effect on viral genome mRNA and protein expression. The selected NS2A segment, a relatively conserved region in JEV ssRNA coding sequence, was measure by real-time RT-PCR. The Kae and Dai inhibitory effect on JEV mRNA at 48 h suggested that they play critical roles in inhibition of viral genome replication. Meanwhile, the effects of 25 µM Kae/Dai on viral E and NS1/NS1' protein detected by western blot showed that Kae and Dai both inhibited protein expression, when compared to the mock-infected group. Moreover, the inhibitory effect of Kae on JEV protein expression in cells treated before infection was more potent than that in cells treated after infection and in cells treated with Dai before and after infection. This further revealed that Kae was a more potent antiviral agent than Dai. The expression levels of NS1 and NS1' proteins were lower than that of E protein in the mock-infected cells, indicating that the results of protein expression are related to virus strain. The obvious suppression of NS1' expression in cells treated with Kae showed that Kae might inhibit the efficiency of the α-1 ribosomal frameshift of JEV. As a control, 7-hydroxyflavone did not exert any influence on expression of E, NS1 and NS1' proteins.

The mechanism by which flavonoids and related isoflavones inhibit virus infectivity has yet to be fully elucidated. A review of the current literature has indicated that flavonoids affect virus binding to cell membranes, entry into the cell, replication, viral protein translation within the host cell, and formation of certain glycoprotein complexes of the virus envelope [8]. At the host cell level, isoflavones, mainly genistein but not daidzein, can affect the induction of certain transcription factors and secretion of cytokines; most of these effects have been attributed to a reduction in protein tyrosine kinase activity [18]. Kae and its derivatives have been tested for their potential antiviral properties including reducing herpes simplex virus-1 and poliovirus infection at 0.4 mM and <10 µM, respectively [19], [20]. Previous research also has shown that Kae possesses antiviral properties against H1N1/H9N2 viruses by inhibiting neuraminidase activity, and that flavonoid activity depends on the position and number of hydroxyl groups on the flavonoid backbone. Activity requires the presence of 4′-OH, 7-OH, C_4_O, and C_2_C_3_ functional groups, but is markedly reduced by the presence of a glycosyl moiety [12], [21], [22]. These intriguing properties were also verified in our research, in which Kae had greater anti-JEV activity than Dai had, owing to its structural characteristics. However, until now, the antiviral properties of Dai have not been described *in vivo* or *in vitro*, which may be attributed to the presence of a glycosyl moiety that influences antiviral activity. Greiner et al. [23] have demonstrated that Dai does not alter virus elimination from serum or affect the immune response of the host in virally challenged pigs. Dai also resulted in activity against JEV in the present study, especially in cells pretreated before infection, suggesting that Dai might share the same binding site of the target molecule with Kae.

Flavonoids are reported to possess the ability to be internalized by platelets and cells. The internalization of Kae observed by confocal microscopy indicated that Kae had high transmembrane permeability in cells. The higher Kae fluorescence observed in infected cells compared to uninfected cells indicates that Kae might bind to JEV RNA. In contrast, no fluorescence was observed in 7-hydroxyflavone- or Dai-treated infected cells or control cells at any time point. In particular, 7-hydroxyflavone was scattered in the extracellular medium, indicating that it has lower transmembrane permeability and viral RNA binding properties than Kae have. Generally, flavonoid activity is attributed to the ability of these compounds to interact with proteins, lipids and polynucleotides through electrostatic and hydrophobic forces, which are necessary for biomolecular interaction within cells and cell–cell interaction, and signal transduction [24], [25]. The current study shows that the high potency of JEV inhibition by Kae, attributed to its structure, may be achieved through attenuation of viral mRNA replication and protein expression. In contrast, Dai may inhibit JEV activities as potently as Kae, involving moderate-potency inhibition of RNA replication and low-potency inhibition of protein expression. Proteins within the same pathway blocked with different potencies by the flavonoids is likely related to internalization of Kae by cells. Therefore, high transmembrane permeability and binding characteristics with JEV RNA of Kae might be related to its antiviral activities.

Several analytical methods have been developed to characterize small ligand interactions with DNA/RNA, including fluorescence spectroscopy, nuclear magnetic resonance, and ultraviolet methods [26]–[28]. To address the molecular basis for the inhibitory mechanisms underlying the effects of Kae and Dai against JEV, flavonoid binding with RNA was further studied by ESI-MS. ESI-MS is a soft ionization process that was used to detect non-covalent interactions [29]. Since then, ESI-MS has been extended to the study of non-covalent interactions between nucleic acids and ligands as a screening tool for drug discovery [30]. ESI-MS has been successful due to the fact that it is a powerful and reliable method that can be used to determine stoichiometry, relative binding affinities with multiple ligands or targets even at very low abundance [31], [32]. Here, the ESI-MS results demonstrated that Kae and Dai were both able to form non-covalent complexes with fsRNA1/fsRNA2, and Kae showed stronger binding affinity with fsRAN1 and fsRNA2 than Dai did. By comparison, ratios of up to 1∶7 and 1∶5 were observed in the case of the fsRNA1–Kae and fsRNA2–Kae complexes, whereas ratios of up to 1∶2 were observed with the fsRNA1–Dai and fsRNA2–Dai complexes. The fsRNA1–ligand complex showed stronger affinities than fsRNA2–ligands, indicating that the affinities may depend on RNA sequence and selectivity of ligands. Furthermore, eight G bases in fsRNA1 and five in fsRNA2 might contribute to the binding sites in accordance with the largest stoichiometric ratio. Electrospray ionization is sufficiently gentle to allow the ionization and detection of intact non-covalent complexes in the gas phase, which depends on sample solution conditions. In this experiment, solvation conditions for RNA and a series of mixtures of RNA and flavonoid compounds were adjusted appropriately (a 5 µM solution of RNA or mixture of 10 µM RNA and 50 µM flavonoid, 150 mM ammonium acetate, containing an equal volume of methanol and water, pH 6.8). Here, methanol was beneficial to dissolution for hydrophobic flavonoids and MS electrospray conditions. When the ratio of flavonoid∶RNA increased from 5∶1 to 10∶1, ratios of up to 1∶8 fsRNA1–Kae complexes were observed. Moreover, the relative abundance of fsRNA1–Kae complexes (1∶1, 1∶2, 1∶3 and 1∶4) increased and that of RNA decreased in MS (Figure S2). As a control, we selected 7-hydroxyflavone, which displayed no anti-JEV activity, to test its affinity to fsRNA1 and fsRNA2. Increasing the ratio of 7-hydroxyflavone∶fsRNA1 to 80∶1 allowed the detection of 1∶2 7-hydroxyflavone∶fsRNA1 complexes, but they had lower relative abundance when compared with Kae–fsRNA1 complexes. We demonstrated that Kae had higher binding affinity to fsRNA1 and fsRNA2 than Dai and 7-hydroxyflavone in the presence of ammonium ions, by using ESI-MS. Therefore, ESI-MS provided proof for RNA binding affinity of Kae/Dai.

In the gas phase, the external medium (vacuum) is widely considered to be hydrophobic. This reinforces the strength of hydrogen bonds compared to hydrophobic and van der Waals interactions [33]. Therefore, one must be cautious in the structural analogy between gas-phase and solution complexes; this is a general debate in the MS of non-covalent complexes [34]. To elucidate this problem further, the binding reactions between RNA and flavonoids in an aqueous phase were studied using ITC. ITC is an important tool for measuring the thermodynamics of interactions between small molecules and biopolymers, in which all binding parameters (n, K, ΔH and ΔS) are simultaneously determined in a single ITC experiment, thus obtaining information that cannot be obtained by other methods [35]–[37]. In our research, analysis of the ITC measurements revealed two interactions to be primarily enthalpy driven. An interesting feature of the thermodynamic data is obvious variation in energies owing to differences in the structure of the bound ligand molecule. Kae was confirmed to have stronger interaction with fsRNA3 (49 nt) compared with that of Dai, which was in accordance with the ESI-MS results. Usually, hydrogen binding, Van der Waals forces, and electrostatic and hydrophobic interactions are the main forces between small molecules and RNA. According to our thermodynamic results, binding of fsRNA3 was exothermic and driven by a moderately favorable enthalpy decrease, in combination with a small entropy change, in the case of Dai. Dai binding to fsRNA3 might be associated with several non-covalent molecular interactions such as intercalation of the chromophore system on binding/intercalation to dsRNA through electrostatic and hydrophobic interactions (ΔH<0, ΔS>0). In the case of Kae, the binding of fsRNA3 was exothermic and driven by favorable enthalpy increase combined with an entropy decrease. It is likely that Kae binding to the fsRNA3 pseudoknot structure occurred through a variety of forces including hydrogen bonding or Van der Waals forces (ΔH<0, ΔS<0), which might have led to considerable distortion of the fsRNA stem-loop structure, and in turn caused disruption of the ordered water structure. Previous reports of binding of some small molecules to tRNA have indicated single binding events. For example, the interaction of an alkaloid complex with tRNA, studied by ITC, suggests that the binding of these alkaloid molecules to the tRNA structure appears to be mostly through partial intercalation [38]. The characterization of fsRNA3 interacting with Kae or Dai has revealed they are involved in diverse stages of the viral life cycle, either replication or virus protein expression.

However, organic solvents (e.g. DMSO or methanol) strongly influence thermodynamic parameters from ITC because of the exothermic reaction. Hence, we cannot acquire thermodynamic parameters for 7-hydroxyflavone and fsRNA under any experimental conditions. Furthermore, it is not worthwhile exploring in detail the hydrophobic 7-hydroxyflavone.

To further research the two flavonoid compounds binding to the frameshift site in a sequence- and/or structure-specific manner, Kae and Dai were docked into RNA using the AutoDock 4.0 program. From three fsRNA docking results (Table 1), the lower BE of Kae complexes with each RNA molecule indicated that Kae could form more stable complexes with three fsRNA than Dai could, which verified the results from ESI and ITC. In particular, the lower BE in complexes of ligands with fsRNA3 molecules compared with fsRNA1/fsRNA2 demonstrated that ligand–fsRNA3 complexes more readily formed non-covalent complexes than ligand–fsRNA1/fsRNA2, which could be attributed to the stem-loop structure of fsRNA3. The highest affinity of Kae–fsRNA3 complexes revealed that Kae could maintain the stability of the stem-loop structure of frameshift RNA. Visualization of the dockings reveals that the Kae and Dai are docked into the minor groove of RNA duplexes, mainly contributing to hydrogen bonds between the ligands and diverse base sequence of RNA, which explains the enhanced antiviral effects of combination treatment with Kae and Dai. Moreover, most hydrogen bonds involve the base G in the RNA sequence. When Kae was docked into another R4 sequence without base G, the BE of the Kae–R4 complex increased to −4.64 kcal mol^−1^, which was elevated by 0.9 kcal mol–1 compared with that of the Kae–fsRNA3 complex. This indicated that dsRNA and the base G in RNA might influence the affinity between Kae and RNA.

To verify the results, the eight dsRNAs sequences selected for the study model were related to a conserved Y CCU UUU slippery heptanucleotide that is found ahead of an RNA pseudoknot structure in an α-1 ribosomal frameshift event. The lowest binding energy (BE, kcal.mol−1) and corresponding energy difference value (ΔE, kcal.mol-1) for each group reflected the ability of RNA to bind ligands Dai and Kae. Compared to the inclusion complexes formed between Kae and dsRNA with UUU triplets, RNA that excluded UUU exhibited the anticipated binding propensity to associate with Kae. We found that Kae binding to RNA without UUU triplets not only had lower BE, but superior probabilities. Correspondingly, another important conclusion can be drawn by comparison with the inclusion complexes of Dai with RNA duplexes without UUU triplets. It may be inferred that the structure of inclusion complexes for Dai binding to RNA molecules without UUU triplets is present in the preferred binding mode as well.

For instance, the interaction energy for the complex of N1a (BE, −4.46 kcal mol−1) decreased markedly compared with that of U1a ((BE, −3.94 kcal mol−1)), corresponding to the lowest BE of −5.64 kcal mol−1 for N1b and −4.83 kcal mol−1 for U1b. This phenomenon might be ascribed to the specificity of the structures of RNA containing UUU triplets and more G bases in the N1 RNA sequence without UUU triplets. Moreover, the specificity remains when initial structures of the selected RNA fragments contain more contiguous U base pairs. Therefore, the eight and nine base pairs are chosen from two RNA fragments, 5′-(UAAAAAAG)2-3′ (U5) and 5′-(GAUUUUUCG)2-3′ (U6), from the second and third RNA sequences given in this work, respectively. For example, the lowest BE of U6b was 0.82 kcal mol−1 lower than that of U6a (EU6a = −3.84 kcal mol−1, EU6b = −4.66 kcal mol−1) even if U5a had a higher frequency of 54% (PU6b = 28%).

Visualization of the docking into the minor groove of RNA duplexes reveals that Kae and Dai mainly bind dsRNA depending on hydrogen bonds between ligands and RNA, indicating preferential binding for Kae or Dai to a stem-loop structure of the frameshift on JEV RNA. Visualization of the dockings also reveals that the top ranked conformation and binding sites are characterized by containing a special sequence of three base (CGG or AGG) when ligands are docked into the RNA. The inclusion complexes of Kae with RNA are more stable than those with Dai, in agreement with the results of ESI-MS and ITC assay, despite the different measurement principles.

Many flavonoids exert their antiviral effects through rings that bind to the viral proteins, or interaction with viral RNA and efficiently blocking viral uncoating. This is the basis of designing new and more efficient antiviral drugs, and their effectiveness depends on the mode and affinity of the binding [13], [39]. It is known that cellular function is often triggered by interactions of flavonoids (including Kae) with dsDNA or tRNA, because Kae exhibits high potential as antioxidant chemotherapy [40], [41]. The antiviral activities of these natural products are attributed to the same mechanism of action. Thus, the difference in binding modes and affinities of Kae and Dai with fsRNA is important in understanding the molecular mechanism of their activities.

It is noteworthy that ESI-MS, ITC and docking data for Kae binding to fsRNA are in excellent agreement with the cell studies. The binding characteristics obtained for Kae and the relation to the frameshift structure of RNA suggest that Kae influences frameshift efficiency in virus-infected cells through interfering with a downstream RNA pseudoknot structure. Furthermore, Kae might affect expression of flaviviral NS1', which is involved in viral neuroinvasiveness. Therefore, the programmed translational frameshift site might be the target for Kae as an antiflaviviral drug.

In this study, we investigated the ability of Kae and Dai, two natural plant extracts, to suppress JEV replication and E viral protein expression in BHK21 cells. Our results suggest that Kae is more promising than Dai for its anti-JEV effects. In addition, further investigations of the binding properties of structurally different flavonoids to RNA showed that Kae possessed relatively stronger binding affinity for RNA and formed non-covalent complexes more easily. Our results may elucidate new aspects of molecular targets for anti-JEV chemotherapeutics and facilitate our understanding of flavonoid transport in cells and prediction of their antiviral activities by focusing on their structural features. The results are helpful for a better understanding of antiviral mechanisms and have broad applications in dietary prevention of viral infectious diseases.

## Materials and Methods

### Reagents

Kae (C_15_H_10_O_6_ XLogP3,1.9; H-Bond Donor, 4; H-Bond Acceptor, 6, *Mr.286.23*) and Dai (C_21_H_20_O_9_, 416.38 g mol^−1^, 3-(4-hydroxyphenyl)-7-[(2S,3R,4S, 5S,6R)-3,4,5- trihydroxy-6-(hydroxymethyl) oxan-2-yl] oxychromen-4-one) were purchased from Sigma (St. Louis, MO, USA). For cell experiments, Kae and Dai were both dissolved in DMSO and finally diluted in culture medium. To avoid toxicity or interference from the solvent, the maximum concentration of DMSO in the medium was <0.1%. A downstream fsRNA pseudoknot structure, a stem-loop structure, (fsRNA3, 5′ CCUUUUCAGCUGGGCCUUCUGGUGAUGUUUCUGGCCACCCAGGAAGUCC3′, 49 nt, 3555–3604 nt) of NS1' ribosomal frameshift event were chemically synthesized and purified by HPLC (Integrated Biotech Solutions Co. Ltd.). The two complementary segments of fsRNA3 were also synthesized using the above methods: fsRNA1 (5′ GGGCCUUCUGGUGAUGUUU 3′, MW, 6048 Da) and fsRNA2 (5′ GGCCACCCAGGAAGUCC 3′, MW, 5423 Da). fsRNA1, fsRNA2 and fsRNA3 were selected to study interaction with Kae or Dai.

### Cells and Viruses

BHK21 cells (ATCC, USA) were propagated and maintained in Dulbecco's modified Eagle's medium (DMEM; Gibco) supplemented with 10% fetal bovine serum (Gibco), 100 U/ml penicillin and 100 µg/ml , streptomycin) at 37°C with 5% CO_2_. The JEV strain SA 14-14-2 (GenBank accession No. AF315119) used in this study was stored in our Department of State Key Laboratory for Molecular Virology and Genetic Engineering (Beijing, China). JEV was propagated in BHK21 cells and stored at −80°C before use.

### Toxicity in BHK21 Cells

Cellular toxicity of Kae and Dai were tested according to a previously reported cell viability assay [42]. Monolayers of BHK21 cells in 96-well plates were incubated with Kae or Dai at concentrations of 5–500 µM in DMEM for 72 h, and 20 µl MTS/PMS(3-(4,5-dimethylthiazol-2-yl)-5-(3-carboxymethoxyphenyl)-2-(4-sulfophenyl)-2H-tetrazolium/phenazine methosulfate: Promega, Madison, WI, USA) was added to each well, and the absorbance at 490 nm was measured according to the manufacturer's recommendations. Percent of cell cytotoxic effect = [1−(At/As)]×100%. At and As indicate the absorbance of the test substances and the solvent control, respectively. The CC_50_ values were defined as the concentration that caused 50% cytotoxicity. Maximum non-toxic dose was determined microscopically by observing morphological changes after 72 h incubation.

### Antiviral Activity Against JEV

Kae and Dai were tested for their antiviral activity against JEV using the MTS assay with monolayer cultures of BHK21 cells grown in DMEM. Cells were infected with 0.1 MOI JEV in 96-wells plates. After 2 h adsorption at 37°C, plates were washed and the medium replaced with DMEM containing 2% fetal bovine serum and different concentrations of Kae and Dai. After 72 h, 20 µl MTS/PMS was added and the absorbance measured at 490 nm test wavelength and 690 nm reference wavelength using a plate reader. Experiments were also performed by incubating with 5–100 µM Kae or Dai for 2 h, and then infecting with 0.1 MOI JEV. After 72 h incubation at 37°C, absorbance was measured with the MTS method as described above.

VIR was calculated as (A_tv_−A_cv_)/(A_cd_−A_cv_)×100%. A_tv_ represents the absorbance of the test compounds with virus-infected cells. A_cv_ and A_cd_ represent the absorbance of the virus and cells control, respectively. The EC_50_ was calculated by regression analysis.

### IFA

BHK21 cells were grown on 24-well plates. Cell monolayers were infected with JEV at 0.1 MOI/0.5 ml for 2 h. The solution was removed and replaced with DMEM containing 50 µM Kae or Dai. At 24 h after virus inoculation at 37°C under 5% CO_2_ atmosphere, the cells were rinsed carefully three times with PBS (pH 7.4) and fixed with 80% acetone for 15 min at room temperature. After washing three times with PBS (pH 7.4), the cells were incubated with JEV E-D3 monoclonal antibody (Beijing Protein Innovation, China) diluted 1∶400 in PBS (pH 7.4) at 37°C for 1 h. After washing with PBS (pH 7.4), the cells were incubated with the secondary antibody DyLigh594-conjugated goat anti-mouse IgG (Jackson, USA) diluted 1∶200 in PBS (pH 7.4) at 37°C for 1 h. After washing with PBS (pH 7.4), the cells were stained with 500 nM DAPI solution for 10 min at room temperature. After washing three times with PBS (pH 8.0), the plates were observed by fluorescence microscopy (Nikon, TE2000-U, Japan).

In the follow-up study, 24-well plates were used to test JEV mRNA and protein expression. The experiment was divided into four groups with four replicates for each compound: (1) mock infection group: mock infection for 48 h without compound treatment; (2) pretreatment group: cell monolayers were pretreated with 25 µM Kae or Dai for 2 h, and then infected with 0.1 MOI JEV for 48 h; (3) pre-infection group: cell monolayers were infected with 0.1 MOI JEV for 2 h and then treated with 25 µM Kae or Dai for 48 h; and (4) normal control group: healthy cells without any treatment.

### Real-time RT-PCR to Test JEV mRNA Expression

The following primers were used: forward, 5′-CCTCCGTCACCATGCCAGT CTTAG-3′ and reverse, 5′-TTCGCCATGGTCTTTTTCCTCTCG-3′ to amplify a 133-bp length in the region of nt 3976–4108 in JEV strain genome sequence, Tm: 56.8°C. This segment was cloned into pGEM-T Easy vector to construct the standard plasmid, used to obtain a standard curve. In the 48 h pretreatment or pre-infection group, viral RNA was extracted from each well by using RNeasy Mini Kit (Qiagen) according to the manufacturer's instructions. An RT reaction was carried out using Superscript First-Strand Synthesis System (Invitrogen) in a 20-µl reaction mixture using 1–2 µg total RNA, according to the manufacturer's protocol. Real time RT-PCR was conducted using ABI Prism 7000 Real-time PCR System (Applied Biosystems). Reactions were performed in a 50 µl volume that contained 2 µl cDNA, 1 µl each primer and 25 µl Power SYBR Green PCR Master Mix (Applied Biosystems). The absolute quantity of viral RNA was calculated by using the standard curve, and melting curve analysis was performed to verify the authenticity of the amplification.

### Western blotting for Expression of JEV E, NS1 and NS1' proteins in BHK21 Cells

BHK21 cells were grown on 24-well plates. Cell monolayers were pretreated with 25 µM Kae or Dai or infected with 0.1 MOI JEV before treatment. After 48 h, JEV E, NS1 and NS1' proteins were detected by western blotting. Harvested cells were washed three times with PBS, lysed on ice in lysis buffer (50 mM Tris–HCl; 150 mM NaCl; 1% NP-40; 1 mgl^−1^ each of aprotinin, pepstatin, and leupeptin; 1 mM each of EDTA, phenylmethylsulfonyl fluoride, dithiothreitol, and sodium fluoride; pH 7.4). The lysates were centrifuged at 10,000× *g* at 4°C for 15 min and the supernatant collected and stored at −80°C until use. The protein content was determined by the Bradford method. Proteins (20 ng) were mixed with electrophoresis buffer, boiled for 5 min, separated on 12% SDS-PAGE, and transferred to nitrocellulose membranes (Amersham). Membranes were blocked with 10% (w/v) non-fat dried milk in PBS with 0.2% (v/v) Tween-20 (PBST) for 1 h, and incubated overnight with primary mouse anti-JEV E-D3 monoclonal antibody (1∶1000; Beijing Protein Innovation, China), β-actin monoclonal antibodies (1∶1000; Santa Cruz Biotechnology, Santa Cruz, CA, USA) and mouse anti-JEV NS1 monoclonal antibody (1∶50; Abcam, Cambridge, MA, USA) diluted in blocking solution at 4°C. Membranes were rinsed three times with PBST, and incubated with the secondary goat anti-mouse IgG conjugated with IRDye700 fluorescein (1∶10,000 dilution; LI-COR, USA) for 1 h at room temperature. Membranes were rinsed twice with PBST and once with PBS again. Subsequent analysis was performed with a Li-COR Odyssey system and quantified using Odyssey infrared imaging software, with β-actin as a loading control.

### Transmembrane Permeability

Cell monolayers grown on 35-mm confocal dishes were infected with 1 MOI JEV for 24 h, and incubated with 50 µM Kae, 100 µM Dai or 0.1% DMSO for 2 h. As a control, cells without JEV infection were treated with 50 µM Kae or 100 µM Dai for 2 h. The supernatant was removed and cells were washed twice with PBS buffer. According to the intrinsic fluorescence of flavonoids, transmembrane permeability of Kae and Dai was assessed using a confocal microscope (TCS SP5; Leica, Germany). Fluorescence was excited at 405 nm with an argon laser and emitted at 480–500 nm. Three dimensional representations of cells were constructed by the generation of sections in the z-dimension and compiled into z-stacks.

### ESI-MS

ESI-MS was utilized to investigate the binding stoichiometry of small molecules to fsRNA1 (R1) and fsRNA2 (R2). Mass spectra were performed on a Thermo Finnigan LTQ Advantage in the negative ion mode. The heated capillary temperature of the electrospray source was set to 250°C, and the voltage on the heated capillary was 11 V. Full scan mass spectra were recorded in an m/z range from 500 to 2000. The relative intensities of the free and bound RNA in the mass spectra were assumed to be proportional to the relative abundances of these species in solution.

### ITC

The binding affinities and enthalpies of the fsRNA3 (R3) and flavonoids relevant to Kae and Dai were measured by an ITC_200_ calorimeter (Microcal). In the ITC experiment, 8 µM Kae or Dai was loaded into the cell with 100 or 200 µM RNA in the titrating syringe, depending on the binding affinities of the compounds. RNA and flavonoids were dialyzed into the same buffer (50 mM Tris-HCl, 150 mM NaCl and 0.1% DMSO, pH 7.3). The titration experiments were performed at 25°C with an initial 0.4-µl injection for 4 s, followed by 20 2-µl injections of 5 s duration. The spacing between injections was 120 s. The stirring speed during the titration was 500 rpm. Equilibrium association constant (Ka) was obtained by fitting experimental data to a “one set of sites” binding model using the Origin 7 software package (Microcal). Correction for the enthalpy of RNA dilution was carried out by subtracting a straight-line linear fit from the last three data points of the titration, after the interaction had reached saturation.

### Computational Methods

The two ligand molecules were docked into the RNA using the AutoDock 4.0 program (Scripps Research Institute) to investigate further the interaction of the two ligands with fsRNA. Each docking was performed twice and each operation screened 250 conformations that could have been advantageous for docking, namely each docking could have 500 preferred conformations. The sequences of the selected three fsRNA (R1, R2 and R3) are shown in reagents part, in which R1 and R2 were two fragments of R3 (two arms of a stem-loop structure in fsRNA). The single-stranded RNA (ssRNA, R4) contained no G base within its sequence (5′-UUUCCUUCUUU UUAUUUUUCUUUCCACCCAUUAAUUCC-3′), as in the control ssRNA. The three dimensional structures of target R1, R2, R3 and R4 were constructed by Accelrys Discovery Studio 2.1. The geometry of the ligand molecules was taken from NCBI database (http://pubchem.ncbi.nlm.nih.gov), which based on X-ray structures. The grid map of 126×64×66 points and a grid-point spacing of 0.558 Å were used during the docking process. During the dock processes, the grid map for R/R3 and R1/R2 were 126×126×126 and 66×126×100 points, and grid-point spacing for R/R_4_, R_1_ and R_2_ were 0.436 Å, 0.525 Å and 0.508 Å, respectively. The initial structures of the selected eight dsRNA fragments were the 20 base pairs shown in Table 3, in which, four RNA sequences contained UUU triplets assigned as U1–U4, and the others without UUU triplets were assigned as N1–N4, generated using the Nucgen program of the Amber package [43]. The ranking functions of the empirical free energies for the docked configurations have been tested for all docking models [44]. Notably, it has been recently reported that AutoDock gives good results for complexes binding nucleic acids [45].

**Table 3:** Selected dsRNA fragments in AutoDock work.

No.	sequence	No.	sequence
U1	5′-(GCCAGUACGUUUCGUGCUGG)_2_-3′	N1	5′-(GUGGGAGUGAAGAGGGUAGU)_2_-3′
U2	5′-(CUAUGCUUUCCUGGCGGCGG)_2_ -3′	N2	5′-(UCAUAAGGAAUCCUGGCUAU)_2_-3′
U3	5′-(GUCAUUUCUGGUCCAUAGGG)_2_-3′	N3	5′-(CCCACAACGAGAAGCGAGCU)_2_-3′
U4	5′-(GGGAUUUGCUCCCUGCUGCA)_2_-3′	N4	5′-(CACGAGAUGGACGAACCAAG)_2_-3′

U1–U4 contain UUU triplets, and N1–N4 have no UUU triplets.

## Supporting Information

Figure S1 Predicted frameshift motif and pseudoknot structure for JEV near the beginning of the NS2A gene.
(TIF)

Figure S2 ESI-MS full-scan spectra of mixtures of fsRNA1 and Kae at diverse molar ratio of flavonoid∶RNA. (A) Mass spectrum offsRNA1 with Kae at 5∶1 molar ratio of Kae∶RNA. The solution was prepared by mixing equal volumes of 50 µM methanol solution of Kae and 10 µM (150 mM ammonium acetate) solution of fsRNA1; fsRNA1 alone, and 1∶1, 2∶1, 3∶1, 4∶1, 5∶1, 6∶1 and 7∶1 complexes are indicated (B) Mass spectrum offsRNA1 with Kae at 10∶1 molar ratio of Kae∶RNA. The solution was prepared by mixing equal volumes of 100 µM methanol solution of Kae and 10 µM (150 mM ammonium acetate) solution of fsRNA1; 1∶1, 2∶1, 3∶1, 4∶1, 5∶1, 6∶1, 7∶1 and 8∶1 fsRNA1/Kae complexes are indicated.
(TIF)
