# Supplementary figures and images for: Anti- Japanese-Encephalitis-Viral Effects of Kaempferol and Daidzin and Their RNA-Binding Characteristics
Source: PLoS One. 2012 Jan 20;7(1):e30259. doi: 10.1371/journal.pone.0030259 (PMC3262791; doi:10.1371/journal.pone.0030259)

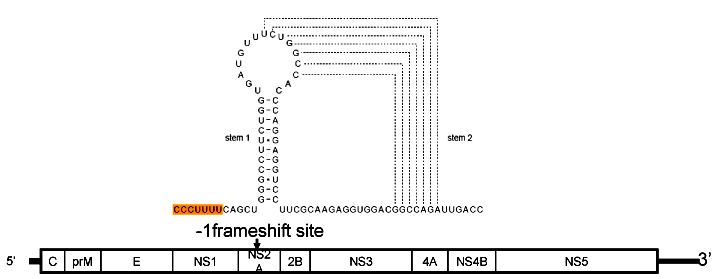

Supplement: Figure S1 — Predicted frameshift motif and pseudoknot structure for JEV near the beginning of the NS2A gene. (TIF) [file pone.0030259.s001.tif]

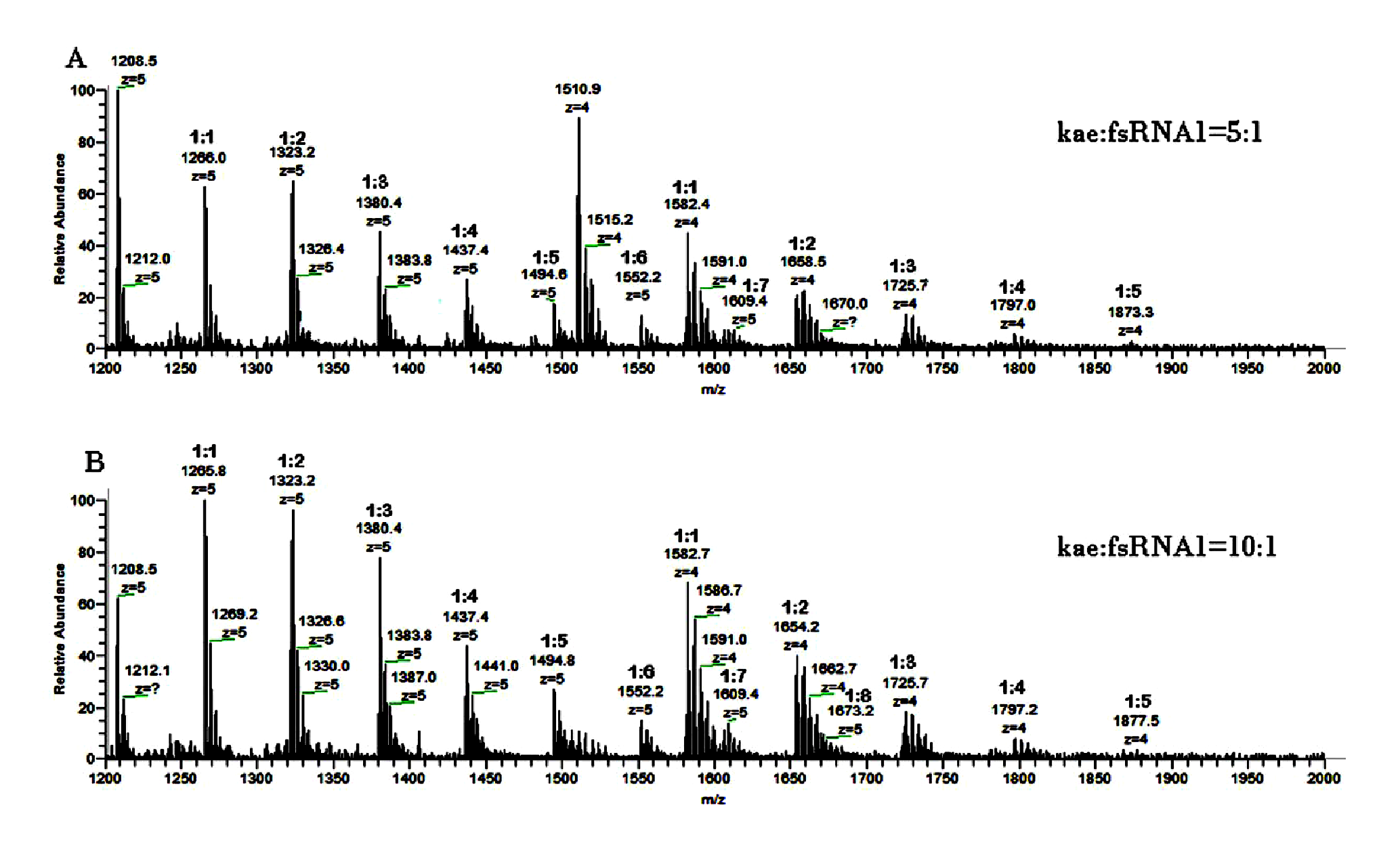

Supplement: Figure S2 — ESI-MS full-scan spectra of mixtures of fsRNA1 and Kae at diverse molar ratio of flavonoid∶RNA. (A) Mass spectrum offsRNA1 with Kae at 5∶1 molar ratio of Kae∶RNA. The solution was prepared by mixing equal volumes of 50 µM methanol solution of Kae and 10 µM (150 mM ammonium acetate) solution of fsRNA1; fsRNA1 alone, and 1∶1, 2∶1, 3∶1, 4∶1, 5∶1, 6∶1 and 7∶1 complexes are indicated (B) Mass spectrum offsRNA1 with Kae at 10∶1 molar ratio of Kae∶RNA. The solution was prepared by mixing equal volumes of 100 µM methanol solution of Kae and 10 µM (150 mM ammonium acetate) solution of fsRNA1; 1∶1, 2∶1, 3∶1, 4∶1, 5∶1, 6∶1, 7∶1 and 8∶1 fsRNA1/Kae complexes are indicated. (TIF) [file pone.0030259.s002.tif]
